# Comment: silent burden no more: a global call to action to prioritize perinatal mental health

**DOI:** 10.1186/s12884-022-04645-8

**Published:** 2022-04-11

**Authors:** Shanon McNab, Jane Fisher, Simone Honikman, Linos Muvhu, Rebecca Levine, Genesis Chorwe-Sungani, Sarah Bar-Zeev, Tedbabe Degefie Hailegebriel, Ifeyinwa Yusuf, Neerja Chowdhary, Atif Rahman, Paul Bolton, Claire-Helene Mershon, Mona Bormet, Diana Henry-Ernest, Anayda Portela, Suzanne Stalls

**Affiliations:** 1 Independent Consultant, Bangkok, Thailand; 2 School of Public Health and Preventive Medicine, Monash University, Melbourne, Australia; 3 Perinatal Mental Health Project, University of Cape Town, Cape Town, South Africa; 4 Society for Pre and Post Natal Services (SPANS), Harare, Zimbabwe; 5 United States Agency for International Development, Washington, DC USA; 6 Kamuzu University of Health Sciences, Blantyre, Malawi; 7 United Nations Population Fund (UNFPA), New York, USA; 8 UNICEF, Headquarter, New York, NY USA; 9 Nigeria Health Watch, Abuja, Nigeria; 10 World Health Organization, Geneva, Switzerland; 11 University of Liverpool, Liverpool, UK; 12 Bill & Melinda Gates Foundation, Seattle, WA USA; 13 Christian Connections for International Health, Nairobi, Kenya; 14 Caribbean Regional Midwives Association, Couva, Trinidad; 15 Department of Maternal, Newborn, Child and Adolescent Health and Aging, World Health Organization, Geneva, Switzerland; 16 MOMENTUM Country and Global Leadership, Washington, DC USA

**Keywords:** Perinatal mental health, Common perinatal mental disorders, Call to action, Women’s health, Child health, Low-and-middle-income countries, Policy, Social determinants of health

## Abstract

Common perinatal mental disorders are the most frequent complications of pregnancy, childbirth and the postpartum period, and the prevalence among women in low- and middle-income countries is the highest at nearly 20%. Women are the cornerstone of a healthy and prosperous society and until their mental health is taken as seriously as their physical wellbeing, we will not improve maternal mortality, morbidity and the ability of women to thrive. On the heels of several international efforts to put perinatal mental health on the global agenda, we propose seven urgent actions that the international community, governments, health systems, academia, civil society, and individuals should take to ensure that women everywhere have access to high-quality, respectful care for both their physical and mental wellbeing. Addressing perinatal mental health promotion, prevention, early intervention and treatment of common perinatal mental disorders must be a global priority.

## Main text

Addressing perinatal mental health (PMH) promotion, prevention, early intervention and treatment of common perinatal mental disorders must be a global priority if we truly want to achieve Sustainable Development Goals 3 (health and well-being) and 5 (gender equality) and improve maternal, newborn, and child health outcomes. Common perinatal mental disorders (CPMDs), consisting of anxiety, depression and somatic disorders, are the most frequent complication of pregnancy, childbirth, and the postpartum period [1, 2].^1^ In low- and middle-income countries (LMICs), prevalence of CPMDs is nearly 20%, and higher among the most marginalized women with the least access to health and social care [1, 3]. Inadequate detection of CPMDs and lack of available services and/or effective referral systems frequently result in women remaining undiagnosed and untreated, suffering in silence while negatively impacting their own quality of life and the physical and emotional health of their children and families.

The traditional biomedical binary construct that a person is either mentally well or ill does not reflect the needs women have along the mental health continuum. CPMDs are complex in origin and expression and social determinants of health such as poverty, harmful gender norms, and intimate partner violence contribute to CPMDs globally, and especially in LMICs [4, 5]. Improving women’s mental health thus requires an intersectoral response that includes government, health sector, social development systems, communities, and families. PMH is therefore not just a ‘woman’s’ problem, but an issue that impacts our very wellbeing as a society.

Women are the cornerstones of a healthy and prosperous society. We must commit now to valuing their physical *and* mental wellbeing, especially during pregnancy and postpartum, and their under-recognized role as primary caregivers for the next generation. The COVID-19 pandemic, which led to increased depression and anxiety in perinatal women, exposed and exacerbated many risk factors that impact women’s health and brought the issue of mental health, and specifically PMH, to the forefront [6–8].

Our urgent call comes on the heels of several global efforts. The 2014 Lancet Commission on Perinatal Mental Health called for the inclusion of PMH in all mental health programs, and the 2018 Lancet Commission on Global Mental Health and Sustainable Development warned that the abysmally slow response to addressing mental health would lead to missing Sustainable Development Goal 3.4 targets explicitly promoting mental health and wellbeing [5, 9]. Global guidelines have elevated these priorities, particularly in LMICs where women are disproportionately impacted [10–12]. In September 2021, a global Maternal Mental Health Technical Consultation convened by the United States Agency for International Development, in collaboration with the United Nations Population Fund and the World Health Organization, with 700 participants from 72 countries, called for urgent action [13]. The message was clear—the challenge is complex but not intractable. Solutions must be evidence-based, involve women to create community-driven solutions, while changing national policy and global standards - and they must include CPMD prevention and mental health promotion. The global community needs to act with urgency, otherwise women, children and families will suffer the consequences: intergenerational poverty, women’s morbidity and mortality, and children not meeting growth and development indicators. To promote perinatal mental wellbeing as a human right, we propose the following actions:

1. The global community must prioritize women’s PMH needs by establishing global standards, guidelines, and strategies to fully integrate PMH into existing programs; and establish and create indicators and monitoring mechanisms to continually assess global progress towards achieving a PMH target [5, 9–12, 14].
2. Governments should be accountable to women’s PMH needs, state their commitment to PMH explicitly in their national policies, and link policies to budgetary allocations that are adequately financed and integrated across other health and development sectors to promote intersectoral collaboration [15]. And governments must also recognize that effective health policies must be aimed at improving the social status of women to have the impact women need and deserve [3].
3. Health systems need to integrate evidence-based PMH approaches into sexual, reproductive, maternal, newborn, child, and adolescent health services; nutrition services; and universal health coverage [16]. The capacity of existing providers should be strengthened through competency-based training, supportive supervision, and adequate remuneration. A critical mass of providers or provider time should be made available, commensurate with the CPMD burden of the local context. Provider skills should include mental health promotion, prevention, and detection; respectful, culturally appropriate person-centered psychological care; and case management.
4. There should be a global commitment to strengthening implementation including implementation research and building on the emerging evidence for practice in LMIC settings with significant learning from the humanitarian sector. There is promising data from several trials that task-sharing approaches and digital interventions are effective for delivery of psychological interventions and treatment by lay health workers [17, 18]. For sustainability, real world implementation strategies need to be developed and assessed where these approaches are adapted for context and fully integrated into health systems at scale, with adequate fidelity. Particular attention should be given to women living in conflict and crisis affected situations, with adaptations made to meet the increasing scale of the need in these contexts.
5. At the community level, evidence-based task sharing models and collaboration with existing platforms and community structures should be employed [19, 20]. Integrated care pathways between community and facilities should be strengthened. Effective strategies to address family issues affecting women’s mental health and reduce stigma should be integrated across these platforms.
6. When social determinants are addressed as part of PMH interventions, women’s overall health and socioeconomic status improves. Interventions to improve PMH should also include solutions outside the traditional health arena: peer support groups, economic empowerment, and intimate partner violence interventions. Given the gendered nature of CPMDs, each of these interventions should be gender intentional and gender transformative [21, 22]. And partners should be included in interventions and acknowledged for the role they can play in PMH.
7. At the individual citizen level, we must work to address the paralyzing shame and stigma preventing women from seeking and accessing care and from having honest conversations about the burden that is impacting their lives and capabilities [23, 24]. Though we recognize that stigma is a social construct and one that must be changed at the societal level, we each have a role as individuals in making these changes in how we think, act and respond

Together, as a global community and as individuals, we can ensure that women everywhere have access to high-quality, respectful care for both their physical and mental wellbeing. If we address the underlying social determinants that predispose women to mental ill health, we will be able to shift the well-being of women and communities. As part of a human rights approach, this must be coupled with a commitment, at all levels of society, to improve the quality and coverage of interventions that address CPMDs. Without this work, we will continue to contribute to the silent burden carried by women and their children. The time for action is now.

## Data Availability

Not applicable.

## Abbreviations

PMH: Perinatal mental health
CPMD: Common perinatal mental disorder
LMIC: Low- and middle-income country

## Footnotes

1 For the purpose of this Commentary the postpartum period includes up to 2 years following childbirth.
